# Editorial: The Science of Pair-Bonding and Future Directions

**DOI:** 10.3389/fpsyg.2020.02178

**Published:** 2020-10-02

**Authors:** Xiaomeng Xu, Bianca P. Acevedo

**Affiliations:** ^1^Department of Psychology, Idaho State University, Pocatello, ID, United States; ^2^Department of Psychological and Brain Sciences, University of California, Santa Barbara, Santa Barbara, CA, United States

**Keywords:** pair bonding, close relationships, romantic relationships, dating, marriage, attachment, evolutionary psychology

The goal of this Research Topic (RT) was to bring together broad scholarship on the science of pair-bonding and to inform future directions for research on this topic. Many who study pair- bonds do so within particular disciplines and/or sub-disciplines, often focusing efforts and contributing to knowledge on specific species, behaviors, conceptualizations, mechanisms, theories, levels of analyses, and more recently large data sets available via the rise of online dating. This Research Topic serves as a launch-pad for broader discussions that enable us to learn from one another, cross-pollinate, strengthen our ideas, and nurture interdisciplinary work that advances research on pair-bonding.

Two articles in this RT present original research that highlights the modern landscape of human pair-bonding. Levy et al. use massive mobile dating data from over 421 million potential matches to investigate dating preferences. Their study showed that similarity of characteristics–such as psychological traits, physical traits, personal choices (i.e., desiring the same relationship type), and shared experiences–predicted dating matches.  Mogilski et al. utilize life history theory (LHT) as a framework to understand the moral stigma associated with consensual non-monogamy (CNM). The authors found that individuals in CNM relationships are more likely to report a fast-life history which is linked to being viewed negatively based on inaccurate beliefs about risk and negative outcomes.

Four articles in this RT examine human pair-bonding with studies that include neuroimaging methodology. Tsapelas et al. examined the attractiveness of relationship alternatives by manipulating self-expansion in two studies (one behavioral, one fMRI) with participants in committed relationships. The studies showed that priming the “need for self-expansion” led to better memory for attractive alternatives with traits dissimilar to their partner, and that priming with partner self-expansion led to less fMRI BOLD responsiveness to attractive alternatives (compared with a love prime and a neutral prime). Azhari et al., examine how personality traits and closeness influence brain responses in the prefrontal cortex (PFC) when viewing social interactions. The authors utilize fNIRS methodology and find differences in PFC based on openness, closeness, and the perceived type of social interaction (romantic partners, siblings, friends). Acevedo et al. explore the neural and genetic correlates of romantic love in newlyweds across the first year of marriage genetic analysis (*AVPR1a* rs3, *OXTR* rs53576, *COMT* rs4680, and *DRD4*-7R), and self-reported relationship quality. Their findings establish the importance of the reward system in romantic love and highlight the biological substrates that facilitate the maintenance of romantic love in early-stage marriages.

Two articles in this RT focus on pair bonding in non-human animals. Potretzke and Ryabinin provide a mini-review of the prairie vole (*Microtus ochrogaster*: a socially monogamous rodent), the neurobiology of pair-bonding, and the effects of addictive substances on pair-bonding in the prairie vole as well as in humans. Savidge and Bales present data from two experiments with titi monkeys (*Plecturocebus cupreus*) investigating responses to separation from attachment figures and adult affiliative behavior. Their research showed that decreased infant locomotor behavior in the presence, as opposed to the absence, of a primary attachment figure was related to decreased anxiety-like behavior in adult pair-mates during a novelty response task. The authors conclude that titi monkeys are an appropriate animal model for attachment research.

Finally, three papers in this RT are reviews or theoretical pieces on human pair-bonding. Prior reviews the literature on behavioral synchrony and highlights how using synchrony as a framework leads to a better understanding of pair-bonding across timescales, contexts, and species. Branand et al. review the literature on the inclusion of the other in the self (IOS), including measurement, predictors, and outcomes of IOS, within the context of long-term monogamous pair-bonds in humans, and other important relationship factors (e.g., positive affect, perspective-taking, self-disclosure, shared excitement). They also review recent theoretical work extending traditional understandings of IOS and propose future directions for IOS research and the self-expansion model. Goetz et al. examine evolutionary mismatch in human mating studies. They suggest that human research participants are often non-representative and WEIRD (Western, Educated, Industrialized, Rich, and Democratic). Goetz et al. identify and review the literature on nine additional mismatch characteristics and highlight the STRANGELY WEIRD-ness of participants (interact with Social media, engage in Temporary relationships, can Relocate with relative ease, have Autonomy in mate choice, are Nulliparous, experience social Group segmentation, are being tested in an Educational setting, have Lots of options, and are Young adults). Their article showcases the importance of considering evolutionary mismatch in mating research and provides recommendations for future studies.

Collectively, the articles in this RT on the science of pair-bonding highlight the complexity of this field. It includes work on the synchrony of behavior among couples, synchronization, and similarity in dating choices, and more complicated issues such as the social stigma attached to consensual non-monogamy. It also explores complex psychological factors in romantic relationships such as “inclusion of others in the self” and the need for self-expansion among individuals for making pair-bonding choices and its influence on the desire for alternative partners. Both human and non-human animal studies in this RT provide scientific advances in understanding the biology of pair-bonding, highlighting the importance of reward as well as more complicated processes, such as intimacy and cognition. These studies highlight both basic and complex processes–such as genetic underpinnings, higher-order cognition, and behaviors–which mediate the proclivity to sustain pair-bonds. In sum, the works in this RT highlight the prevailing importance of pair-bonds in humans and other species as they influence biology, behavior, emotions, psychological processes, and complicated processes such as the perception of merging with another and the motivation to expand the self. This RT also highlights the need to widen the scope of practical knowledge to include diverse samples of individuals in relationship studies. Collectively, these works show us that relationship science has made marked strides in advancing our understanding of the complexities of the basic nature of pair-bonds, and they also provide important considerations for future research.

## Author Contributions

Both authors have contributed to the writing of this editorial.

### Conflict of Interest

The authors declare that the research was conducted in the absence of any commercial or financial relationships that could be construed as a potential conflict of interest.

